# FasL Modulates Expression of *Mmp2* in Osteoblasts

**DOI:** 10.3389/fphys.2018.01314

**Published:** 2018-09-19

**Authors:** Eva Svandova, Barbora Vesela, Hervé Lesot, Jeremy Sadoine, Anne Poliard, Eva Matalova

**Affiliations:** ^1^Department of Physiology, University of Veterinary and Pharmaceutical Sciences, Brno, Czechia; ^2^Laboratory of Molecular Morphogenesis, Institute of Animal Physiology and Genetics, The Czech Academy of Sciences, Brno, Czechia; ^3^Faculté de Chirurgie Dentaire, Université Paris Descartes, Paris, France

**Keywords:** osteogenesis, intramembranous bone, Fas ligand, non-apoptotic, *Mmp2*

## Abstract

FasL is a well-known actor in the apoptotic pathways but recent reports have pointed to its important novel roles beyond cell death, as observed also for bone cells. This is supported by non-apoptotic appearance of FasL during osteogenesis and by significant bone alterations unrelated to apoptosis in FasL deficient (*gld*) mice. The molecular mechanism behind this novel role has not yet been revealed. In this report, intramembranous bone, where osteoblasts differentiate directly from mesenchymal precursors without intermediary chondrogenic step, was investigated. Mouse mandibular bone surrounding the first lower molar was used as a model. The stage where a complex set of bone cells (osteoblasts, osteocytes, osteoclasts) is first present during development was selected for an initial examination. Immunohistochemical staining detected FasL in non-apoptotic cells at this stage. Further, FasL deficient vs. wild type samples subjected to osteogenic PCR Array analysis displayed a significantly decreased expression of Mmp2 in *gld* bone. To examine the possibility of this novel FasL–Mmp2 relationship, intramembranous bone-derived osteoblastic cells (MC3T3-E1) were treated with anti-FasL antibody or rmFasL. Indeed, the FasL neutralization caused a decreased expression of *Mmp2* and rmFasL added to the cells resulted in the opposite effect. Since *Mmp2*^-/-^ mice display age-dependent alterations in the intramembranous bone, early stages of *gld* mandibular bone were examined and age-dependent phenotype was confirmed also in *gld* mice. Taken together, the present *in vivo* and *in vitro* findings point to a new non-apoptotic function of FasL in bone development associated with *Mmp2* expression.

## Introduction

FasL (CD178; CD95L; APO1L and TNF ligand superfamily member 6) belongs to the tumor necrosis factor (TNF) family and interacts with Fas (CD95; APO-1; TNFRSF6) receptor (Suda et al., 1993). The Fas/FasL pathway is a well-known regulator of apoptotic cell death, particularly in the immune system (Suda et al., 1993; Griffith et al., 1995; Tsutsui et al., 1996). However, recent evidences have also suggested important non-apoptotic functions of FasL, beyond the immune system, such as in cell proliferation, differentiation or survival, as for instance in bones (Josefsen et al., 1999; Kovacic et al., 2007; Rippo et al., 2013).

Bone development proceeds through an intramembranous or endochondral ossification process. Ossification in the former is direct, while, in the latter, a step of cartilaginous differentiation takes place (Franz-Odendaal, 2011). The involvement of FasL in osteogenesis was suggested from the knock-out bone phenotype in FasL deficient (*gld*) adult mice, which display an increased bone mineral density (BMD) in endochondral (long) bones (Katavic et al., 2003). This has made of FasL a potential target for the treatment of osteoporosis (Park et al., 2005; Nakamura et al., 2007; Kovacic et al., 2010).

In the context of bone formation and homeostasis, Fas/FasL system has been so far connected with osteoblast apoptosis of human biopsies (Kawakami et al., 1997), osteoblasts-induced osteoclast apoptosis in cells derived from long bones (Wang et al., 2015) and was reported along with other apoptosis-associated factors in the human mandible (Hatakeyama et al., 2000). Regarding non-apoptotic functions of FasL in osteogenesis, the Fas/FasL system was shown to influence *in vitro* differentiation of murine endochondral bone derived cells but the mechanism remains unclear (Kovacic et al., 2007).

This work aimed to investigate molecular mechanism(s) behind non-apoptotic osteogenic functions of FasL during intramembranous osteogenesis, where data are lacking. For this purpose, the mandibular bone adjacent to the first molar was selected as a model. Immunohistochemistry, PCR Array-based screening and FasL deficient vs. wild type mouse bone examination were performed *in vivo*. These analytical approaches were completed by *in vitro* functional tests using a cell line to demonstrate a new relation between FasL and Mmp2 in osteoblasts.

## Materials and Methods

### Biological Material

Mice (*Mus musculus*) of the ICR strain were studied at embryonic (E) stages (E13, E15). The FasL-deficient mice (*gld*) strain: B6Smn.C3-*Fasl^gld^*/J and the corresponding controls (C57BL/6J) were purchased from The Jackson Laboratory (Maine, United States) and staged (E15 and P24). Mice were kept in the animal facilities of the Institute of Animal Physiology and Genetics, The Czech Academy of Sciences, v.v.i., Czech Republic and Faculté de Chirurgie Dentaire, Université Paris Descartes, France. The experimental protocol is part of the project GACR 16-18430S and runs under Animal Care Ethics Committee of Université Paris Descartes in France. A cell line of osteoblastic precursors, MC3T3-E1, was purchased from the European Collection of Cell Culture (c.n. 99072810).

### Histological Staining, Immunohistochemistry, Immunofluorescence

Mouse heads were fixed in 4% PFA 24 h. Further, samples were dehydrated in alcohol series, treated with xylene and embedded in paraffin. Histological sections of mandibles (5 μm) were then prepared. Morphological criteria were analyzed by trichrome, haematoxylin-eosin staining or TRAP analysis as was used in Svandova et al. (2018).

For immunohistochemical detection, antigen retrieval was carried out in citrate buffer (pH = 6.0) for 5 min at 98°C in case of osteocalcin. The following primary antibodies were applied: FasL (FasL/N-20, sc-834, Santa Cruz Biotechnology, United States), osteocalcin (Rb pAb to Osteocalcin, ab93876, Abcam, United Kingdom). The primary antibodies were diluted 1:50 (FasL) and 1:100 (Osteocalcin) and applied overnight at 4°C. Peroxidase-conjugated streptavidin-biotin system (Vectastain, United States) and chromogen substrate diaminobenzidine (DAB, K3466; Dako, United States) reactions were used to visualize the positive cells as brown. Slides were counterstained with haematoxylin.

Immunofluorescent analysis of Sost, Mmp2 and FasL expression was applied for mandibles and MC3T3-E1. For Sost and Mmp2, retrieval of antigens was performed. The following primary antibodies were applied: Sost (Mouse Sost/Sclerostin Antibody, AF1589, R&D Systems, United States), Mmp2 (Mmp2/H-76, sc-10736, Santa Cruz Biotechnology, United States) and FasL (described above). Antibodies were diluted 1:50 (Mmp2 and FasL) or 1:100 (Sost) and applied overnight at 4°C. Then Alexa Fluor^®^ 488 (Thermo Fischer Scientific, United States) or Alexa Fluor^®^ 594 (Abcam, United Kingdom) were used (1:200) for 40 min at RT.

Cytoskeleton was visualized by ActinGreen^TM^ 488 ReadyProbes^TM^ Reagent (Thermo Fischer Scientific, United States), nuclei were detected by ProLong^®^ Gold Antifade reagent with DAPI (Thermo Fischer Scientific, United States).

### TUNEL Assay

Histological sections were rehydrated. Samples were pre-treated with 20 mg/ml proteinase K for 15 min at RT. Endogenous peroxidase was blocked by incubating with 3% hydrogen peroxide in PBS for 5 min at RT. The reaction mixture (TUNEL, S7100; Millipore, United States) consisting of 1 μl TdT enzyme: 14 μl distilled water: 35 μl reaction buffer, was prepared and samples were incubated for 45 min at 37°C. Anti-digoxigenin-peroxidase reaction was applied for 30 min at RT. Positive cells were finally visualized by DAB. Samples were counterstained with haematoxylin.

### Analysis of Osteoclast Number and Bone Area

Six samples (three *gld* mice and three wild-type mice, prenatal stage 15) were used for the analysis. The bone surrounding the first molar was investigated to preserve homogeneity of the selected bone region. Five series of four slides (four histological sections per one slide) were prepared for each sample. The first slide of each series was used for morphological observation (haematoxylin-eosin staining), the second for detection of osteoclasts (TRAP), the third for bone matrix visualization (trichrome staining) and the fourth for apoptosis detection (TUNEL). One section per slide was evaluated to avoid multiple counting.

For the osteoclasts analysis, TRAP-positive cells were counted using Fiji application (ImageJ)^1^ in the left and right quadrants of the mandibular bone. Ten values were obtained for each animal to determine the total number of osteoclasts in the region.

The bone matrix area was measured in the trichrome-stained slides. Again 10 values were used to calculate the total area for each specimen. The region of the mandibular bone was expressed in pixels using Adobe Photoshop 6 (Adobe Systems) and recalculated into square micrometers. The average (avg) and standard deviation (SD) was counted for both groups (*gld* and wild type) and statistical significance was determined (Student’s *t*-test, *p* ≤ 0.05).

### Micro Computed Tomography (MicroCT)

Specimens were scanned using a Quantum FX Caliper device (Life Sciences, Perkin Elmer, United States). The skeleton was acquired using an X-ray tube voltage of 90 kV and a tube current of 160 μA with a field of view 10 × 10 mm. Full 3D high-resolution raw data were obtained by rotating both the X-ray source and the at panel detector 360° around the sample (scanning time of 3 min). Projections were reconstructed by Rigaku software in image blocks of 512 × 512 × 512 voxels with an anisotropic voxel size of 20 μm. Data were stored in Dicom frame. Quantification of the jaw bone microarchitecture below the first molar was made by a CTscan Analyzer (Skyscan, release 1.13.5.1, Belgium). The region of interest (ROI) was drawn in the axial sections along the cortical zone between the first and second mesial roots. The bone was segmented in the ROI, and the following parameters were calculated using the CTAnalyzer: bone volume/tissue volume (BV/TV), trabecular thickness (Trab.Th), trabecular number (Trab.N), trabecular pattern factor (Trab.Pf), and BMD. An internal density phantom, calibrated as in mg/cm^3^ of hydroxyapatite, was used to scale the bone density. Selected nomenclature was described in Bouxsein et al. (2010).

Twelve animals at stage P24 were used, six *gld* and six wild-type mice, with both groups consisting of animals of mixed gender. The size of the group was arranged to reach the statistical significance (at least for *p* ≤ 0.05). The averages and standard deviations were determined for all data sets. Normality of the data was tested by Shapiro-Wilks test. Data with a normal distribution (BV/TV, Trab.Th and BMD) were tested by Studen’s *t*-test (two-tailed), and other data (Trab.N and Trab.Pf) were tested by Mann–Whitney test.

### FasL/Anti-FasL Application in MC3T3-E1 Cells

MC3T3-E1 cells were cultured in MEM Alpha medium (Gibco, United States) containing 10% fetal calf serum (FCS) and penicillin/streptomycin (1,000 U/ml, 100 μg/ml). Recombinant Mouse (rm) FasL/TNFSF6 protein (526-SA, R&D Systems, United States) was diluted in a solution of 0.1% FCS in PBS at a final concentration 1 μg/ml. Mouse FasL/TNFSF6 antibody (anti-FasL) (MAB5262, R&D Systems, United States) was diluted in PBS and used in final dilution 20 μg/ml. The dose was determined according to producer recommendation and previously published experiments by Kovacic et al. (2007). Cells were seeded at 5,000 cells/cm^2^ and cultured with rmFasL/anti-FasL for 3 or 6 days simultaneously with control cultures. Cells were harvested using RLT buffer (Qiagen, United States) for further RNA isolation by RNeasy Mini Kit (74106, Qiagen, United States).

### PCR Array

The heads of *gld* mice and wild-type mice (E15) collected into RNAlater (Ambion, United States) were washed in PBS, fixed in 4% paraformaldehyde, and histologically processed in paraffin. Sections (5 μm) were prepared, the mandibular bone was separated and RNA was extracted using RNeasy FFPE Kit (73504, Qiagen, United States) as described by Svandova et al. (2014).

Expression of osteogenic genes was assessed by a PCR Array. RNA was transcribed into cDNA and used for the PCR Array (Mouse Osteogenesis, PAMM-026Z; Qiagen, United States). Data (*n* = 6) were evaluated by Qiagen Data Analysis Center available at https://www.qiagen.com/kr/shop/genes-and-pathways/data-analysis-center-overview-page/. The software provides fold regulation values and statistical significance of the results (*p* ≤ 0.05). The threshold of fold regulation was established as ±2, representing a decrease/increase of 100% in the investigated sample.

The panel of genes in osteogenic PCR Array includes: Acvr1 (Activin A receptor, type I), Ahsg (Alpha 2-HS Glycoprotein), Alpl (Alkaline Phosphatase, Liver/Bone/Kidney), Anxa5 (Annexin A5), Bglap (Osteocalcin), Bgn (Biglycan), Bmp1 (Bone Morphogenetic Protein 1), Bmp2, Bmp3, Bmp3b/Gdf10 (Growth Differentiation Factor 10), Bmp4, Bmp5, Bmp6, Bmp7, Bmpr1a (Bone Morphogenetic Protein Receptor Type 1A), Bmpr1b, Bmpr2, Cd36 (Thrombospondin Receptor), Cdh11 (Cadherin 11), Chrd (Chordin), Col1a1 (Collagen Type I Alpha 1 Chain), Col1a2, Col2a1, Col3a1, Col4a1, Col5a1, Col10a1, Col14a1, Comp (Cartilage Oligomeric Matrix Protein), Csf1/Mcsf (Colony Stimulating Factor 1/Macrophage Colony Stimulating Factor 1), Csf2/GM-CSF (Colony Stimulating Factor 2), Csf3/GCSF (Colony Stimulating Factor 3), Ctsk (Cathepsin K), Dlx5 (Distal-Less Homeobox 5), Egf (Epidermal Growth Factor), Fgf1 (Fibroblast Growth Factor 1), Fgf2/bFGF, Fgfr1 (Fibroblast Growth Factor Receptor 1), Fgfr2, Flt1 (Fms Related Tyrosine Kinase 1), Fn1 (Fibronectin 1), Gli1 (GLI Family Zinc Finger 1), Igf1 (Insulin Like Growth Factor 1), Igf1r (Insulin Like Growth Factor 1 Receptor), Ihh (Indian Hedgehog), Icam1 (Intercellular Adhesion Molecule 1), Itga2 (Integrin Subunit Alpha 2), Itga2b (Integrin Subunit Alpha 2b), Itga3, Itgam (Integrin Subunit Alpha M), Itgav (Integrin Subunit Alpha V), Itgb1 (Integrin Subunit Beta 1), Mmp2 (Matrix Metallopeptidase 2), Mmp8, Mmp9, Mmp10, Nfkb1 (Nuclear Factor Kappa B Subunit 1), Nog (Noggin), Pdgfa (Platelet Derived Growth Factor Subunit A), Phex (Phosphate Regulating Endopeptidase Homolog X-Linked), Runx2 (Runt Related Transcription Factor2), Serpinh1 (Serpin Family H Member 1), Smad1 (SMAD Family Member 1), Smad2, Smad3, Smad4, Smad5, Sost (Sclerostin), Sox9 (SRY-Box 9), Sp7 (Osterix), Spp1 (Osteopontin), Tgfb1 (Transforming Growth Factor Beta 1), Tgfb2, Tgfb3, Tgfbr1 (Transforming Growth Factor Beta Receptor 1), Tgfbr2, Tgfbr3, Tnf (Tumor Necrosis Factor), Tnfsf11 (TNF Superfamily Member 11), Twist1 (Twist Family BHLH Transcription Factor 1), Vcam1 (Vascular Cell Adhesion Molecule 1), Vdr (Vitamin D Receptor), Vegfa (Vascular Endothelial Growth Factor A), Vegfb.

### qPCR

qPCR was performed in 10 μl final reaction volumes containing the one-step master mix gb Ideal PCR Master Mix (Generi Biotech, Czech Republic) using LightCycler 96 (Roche, Switzerland) with preheating at 95°C for 10 min, followed by 40 cycles of 95°C/15 s and 62°C/1 min. *Mmp2* expression levels (Mouse *Mmp2*, Mm00439498_m1, TaqMan Gene Expression Assay, Thermo Fischer Scientific, United States) were calculated using the ΔΔCT method, with normalization against actin levels (Mouse *Actb*, Mm02619580_g1, TaqMan Gene Expression Assay, Thermo Fischer Scientific, United States), which was used as the internal control. For both groups, analysis was performed in three biological replicates, reactions were performed in triplicates for each sample for statistical analysis (Student’s *t*-test, *p* ≤ 0.05).

## Results

### FasL Is Expressed by Non-apoptotic Osteoblasts in Prenatal Mandibular Bone

**FIGURE 1:**
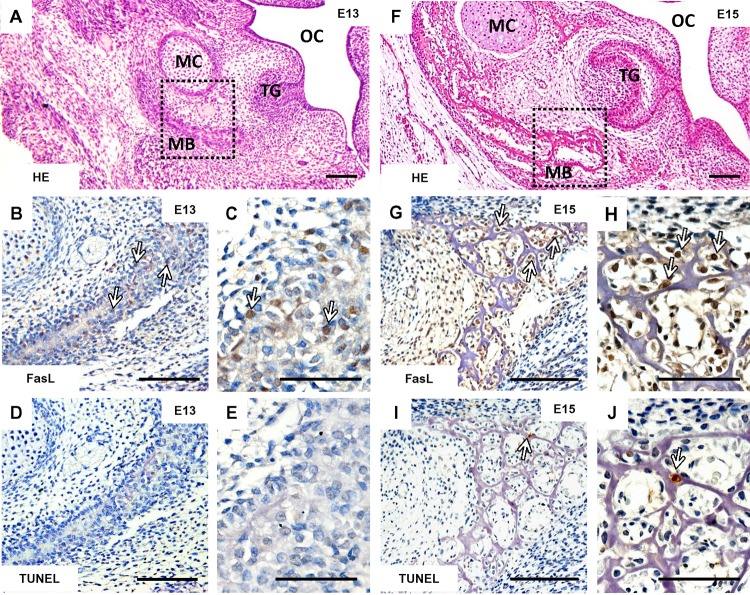
Temporo-spatial analysis of FasL expression and apoptosis. Morphology of the mandible visualized by haematoxylin-eosin staining at E13 **(A)** and E15 **(F)**, immunohistochemical detection of FasL expression in the mandibular bone at E13 **(B,C)** and E15 **(G,H)**, localization of apoptotic cells (TUNEL) in the mandibular bone at E13 **(D,E)** and E15 **(I,J)**. Arrows point to the positive cells (immunohistochemistry-brown). Mandibular bone (MB), Meckel’s cartilage (MC), tooth germ (TG), oral cavity (OC). Scale bar = 100 μm **(A,B,D,F,G,I)**, scale bar = 50 μm **(C,E,H,J)**.

FasL (**Figures 1B,C**) was present already at stage (E13) of the mandibular bone formation (**Figure 1A**) committed to produce osteoblasts. At this stage, only random apoptotic cells can be observed within the mandible (**Figures 1D,E**). At E15, the stage when the first complex set of bone cells (osteoblasts, osteocytes, osteoclasts) appears in the mouse mandibular bone related to the first molar development (**Figure 1F**), FasL displayed a much broader distribution (**Figures 1G,H**) than apoptosis (**Figures 1I,J**).

**FIGURE 2:**
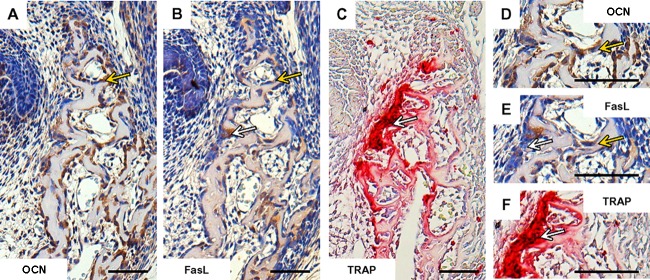
Correlation of FasL expression with markers of bone cells in prenatal (E15) mandibular bone. Distribution of osteocalcin-positive cells/osteoblasts **(A,D)**, localization of FasL expression **(B,E)**, localization of TRAP-positive cells/osteoclast **(C,F)**. Positive cells (immunohistochemistry-brown, TRAP-purple), white arrows point to the osteoclasts, yellow arrows point to the osteoblasts. Scale bar = 50 μm.

Taken together, at the early stage of the mandibular bone formation, FasL expression (**Figures 2B,E**) did not overlap with apoptosis pattern but strongly correlated with osteocalcin expressing cells/osteoblasts (**Figures 2A,D**). FasL expression also corresponded with some TRAP positive cells/osteoclasts (**Figures 2C,F**).

### FasL Deficiency Impacts Osteogenic Markers in the Mandibular Bone

**FIGURE 3:**
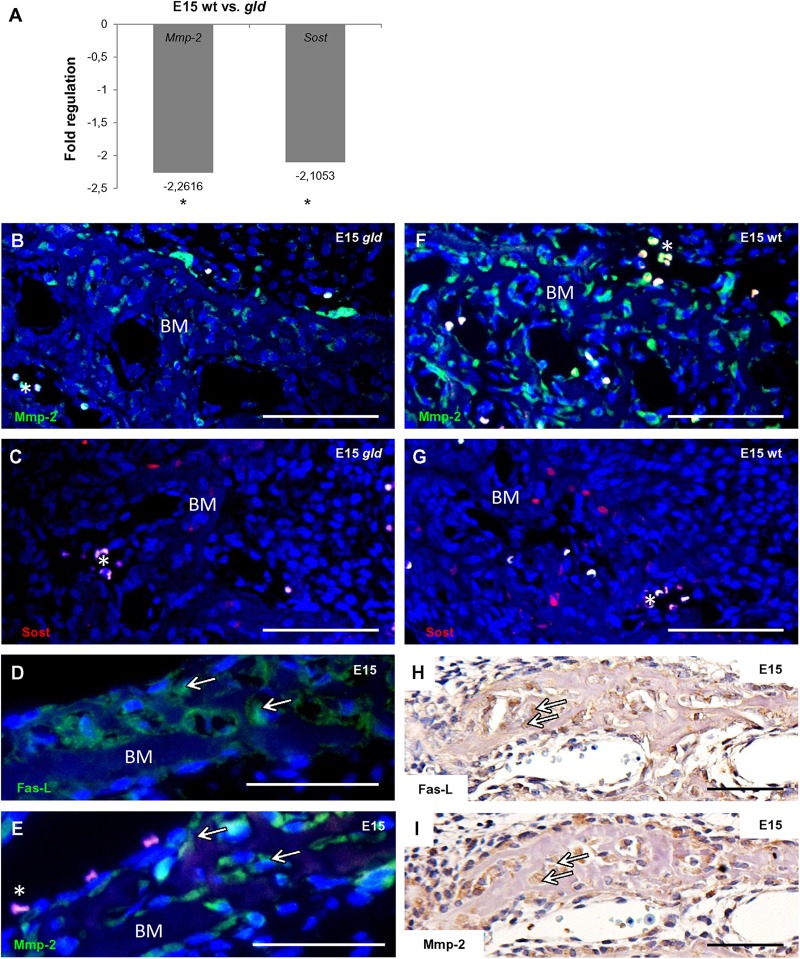
Influence of FasL in expression of osteogenic markers at E15. Decrease in mRNA level of two osteogenic factors (*Mmp2, Sost*) in *gld* mice (*n* = 3 animals) compared to wild types (*n* = 3 animals), *Mmp2* fold regulation -2.262, *p* = 0.0205, *Sost* fold regulation = -2.105, *p* = 0.0235, Student’s *t*-test, two-tailed **(A)**, immunofluorescent detection of Mmp2 in *gld* mouse **(B)** and wild type **(F)**, immunofluorescent detection of Sost in *gld* mouse **(C)** and wild type **(G)**. Correlation of FasL **(D,H)** and Mmp2 **(E,I)** expression. Arrows point to cells expressing simultanously FasL and Mmp2. Mmp2 positive cells are green, Sost positive cells are red, nuclei are blue. Autofluorescence of blood cells is marked by asterisk. Bone matrix (BM). Scale bar = 100 μm **(B–G)**, scale bar = 50 μm **(H,I)**.

In order to find out if FasL deficiency can impact osteogenic pathways in the forming mandibular bone, a PCR Array analysis allowing screening of 84 osteogenic genes was performed. Samples from the mandibular bone obtained from *gld* and wild type mice at E15 were used for comparison. In *gld* mice, the results revealed a statistically significant decrease of the expression of two genes (**Figure 3A**): matrix metalloproteinase 2 (*Mmp2*, fold regulation = -2.262, *p* = 0.0205) and sclerostin (*Sost*, fold regulation = -2.105, *p* = 0.0235).

These results were further validated at the protein level by an immunofluorescence analysis also showing a decreasing trend in the mutant vs. the wild type mice for the Mmp2 (**Figures 3B,F**) and Sost proteins (**Figures 3C,G**). Besides, immunohistochemical staining performed on serial sections of E15 wild type mandibular bone allowed visualization of individual bone cells expressing both FasL (**Figures 3D,H**) and Mmp2 proteins (**Figures 3E,I**).

### Experimental Modulation of FasL Impacts the Expression of *Mmp2* in Osteoblastic Cells *in vitro*

**FIGURE 4:**
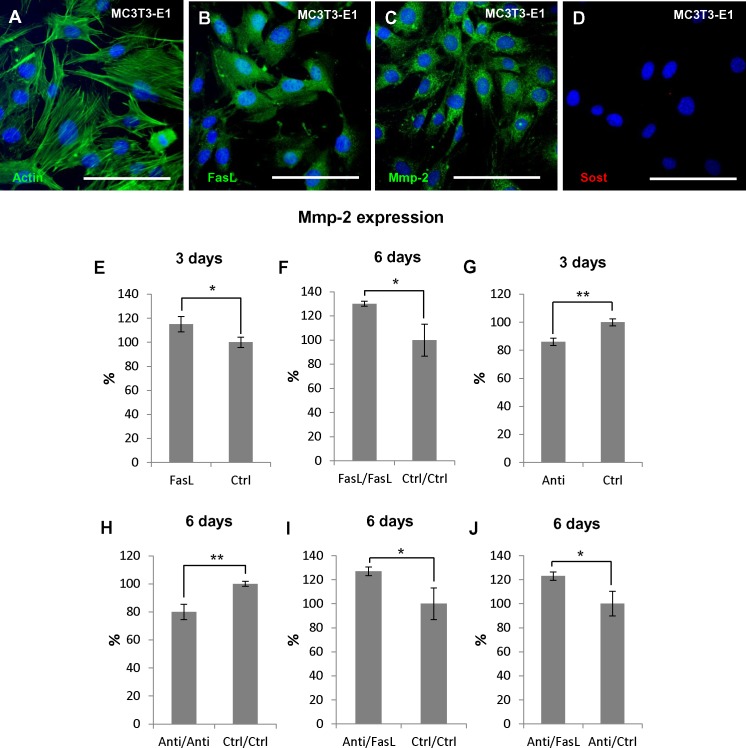
Regulation of *Mmp2* expression in osteoblastic precursors (MC3T3-E1) using rmFasL/anti-FasL. Immunoflourescent detection of actin filaments **(A),** FasL **(B)**, Mmp2 **(C)**, and Sost **(D)** in MC3T3-E1 cells. Actin filaments, FasL, and Mmp2 positive cells are green, nuclei are blue. Expression of *Mmp2* (mRNA) after: 3 days of rmFasL application compared to untreated cells resulted in 15% increase, *p* = 0.0295 **(E)**, 6 days of rmFasL application compared to untreated cells resulted in 30% increase, *p* = 0.0210 **(F)**, 3 days of anti-FasL application compared to untreated cells resulted in 14% decrease, *p* = 0.0024 **(G)**, 6 days of anti-FasL application compared to untreated cells resulted in 20%, *p* = 0.0048 **(H)**, 3 days of anti-FasL application/3 days of rmFasL treatment compared to untreated cells resulted in 27% increase, *p* = 0.0303 **(I)**, 3 days of anti-FasL application/3 days of rmFasL treatment compared to 3 days of anti-FasL application/3 days of untreated culture resulted in 23% increase, *p* = 0.0262 **(J)**. For each group *n* = 3. Scale bar = 100 μm. ^∗^*p* ≤ 0.05 and ^∗∗^*p* ≤ 0.01.

MC3T3-E1 cell line derived from murine calvaria (intramembranous) bone (**Figure 4A**) that naturally produces FasL (Ozeki et al., 2002; **Figure 4B**) as well as Mmp2 (Mizutani et al., 2001; **Figure 4C**) were used for the functional experiments where alterations of *Mmp2* expression (mRNA levels) after FasL stimulation or inhibition were followed. Sost is not expressed by MC3T3-E1 (**Figure 4D**).

*Mmp2* expression was evaluated after addition of recombinant FasL (rmFasL) to the culture medium, or endogenous FasL inhibition by anti FasL antibodies. Addition of rmFasL to MC3T3-E1 resulted in up to a 15% (*p* = 0.0295) increase in *Mmp2* expression in comparison to control samples as soon as after 3 days of culture (**Figure 4E**). This increase was even higher after 6 days (30%, *p* = 0.0210) (**Figure 4F**). Conversely, cell treatment with anti-FasL antibodies caused up to a 14% decrease in *Mmp2* expression (*p* = 0.0024) after 3 days of culture (**Figure 4G**), and a 20% decrease (*p* = 0.0048) after 6 days (**Figure 4H**).

Additionally, combinatory experiments were performed to evaluate a possible rescue effect of the treatments: 3 days anti-FasL + 3 days rmFasL led to a 27% increase in *Mmp2* expression when compared to 6 days untreated (*p* = 0.0303) (**Figure 4I**); 3 days anti-FasL + 3 days rmFasL led to a 23% increase in *Mmp2* expression when compared to 3 days anti-FasL + 3 days untreated (*p* = 0.0262) (**Figure 4J**).

These additional *in vitro* experiments further confirmed the modulatory effect of FasL on Mmp2 expression as identified by PCR Arrays from *in vivo* dissected bone samples.

### *Gld* Jaw Bones Show Age Dependent Alterations in Bone Phenotype

**FIGURE 5:**
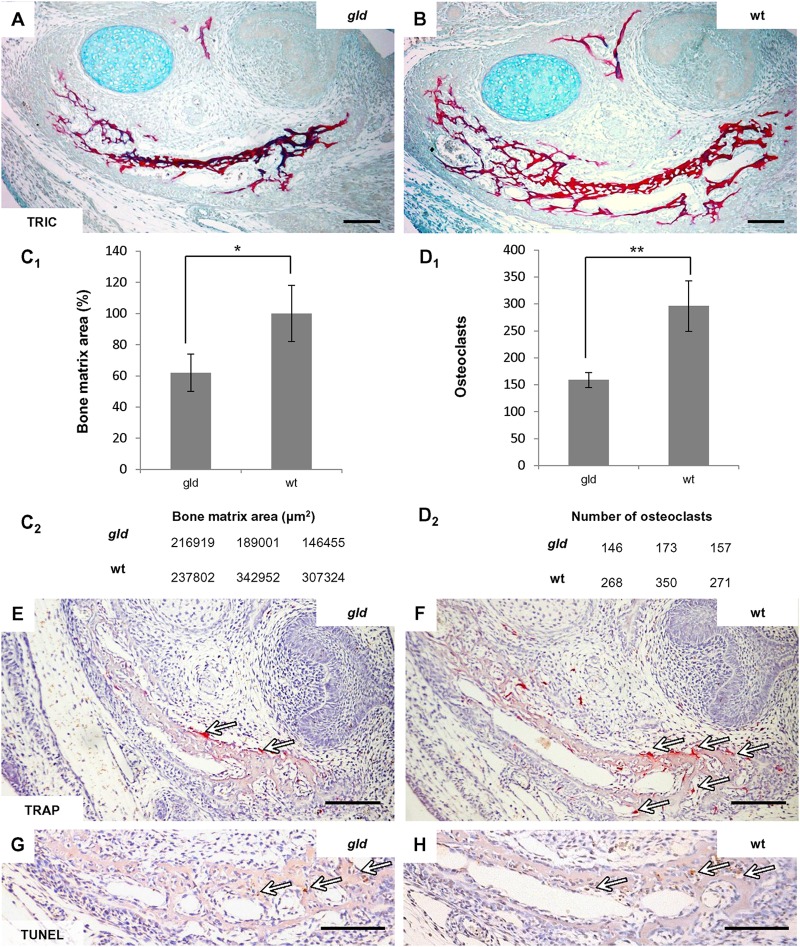
Analysis of prenatal (E15) jaw bone of *gld* mice. The mandibular bone visualized by trichrome staining in *gld* mouse **(A)** and wild-type mice **(B)**, decrease of bone matrix in *gld* mice (*n* = 3 animals) compared to wild-type animals (*n* = 3 animals), avg*_gld_* = 184.125 μm^2^, sd*_gld_* = 35.484, avg_wt_ = 296.026 μm^2^, sd_wt_ = 53.478, *p* = 0.0392 **(C_1_,C_2_)**, decrease of osteoclasts detected by TRAP-staining in *gld* mice (*n* = 3 animals) compared to wild-type mice (*n* = 3 animals), avg*_gld_* = 159, sd*_gld_* = 14, avg_wt_ = 296, sd_wt_ = 47, *p* = 0.0079 **(D_1_,D_2_)**, TRAP-staining in *gld* **(E)** and wild-type mice **(F)** detection of apoptotic cells (TUNEL) in the mandibular bone of *gld* **(G)** and wild-type mice **(H)**. Arrows point to the positive cells (immunohistochemistry-brown, TRAP-purple). Scale bar = 100 μm. ^∗^*p* ≤ 0.05 and ^∗∗^*p* ≤ 0.01.

In order to search for similarities in FasL and Mmp2 deficient mice, published data (Inoue et al., 2006) reporting an age-dependent phenotype in Mmp2^-/-^ intramembranous bones were supplemented by analysis of jaw bones in the *gld* mice in prenatal and young adults. At E15, the stage subjected to PCR Array analysis, the mandibular bone of the *gld* mice showed an abnormal bone microarchitecture, which appeared to be underdeveloped as compared to the bones of wild-type mice (**Figures 5A,B**). Indeed, the area of mandibular bone matrix was decreased by 38%, *p* = 0.0392 (**Figures 5C_1_,C_2_**) in *gld* mice (avg*_gld_* = 184.125 μm^2^, sd*_gld_* = 35.484) compared to the wild-type mice (avg_wt_ = 296.026 μm^2^, sd_wt_ = 53.478). This decrease in bone area did not appear to result from an increase in the osteoclast population (**Figures 5E,F**) since the osteoclast number, in the mandibular bone adjacent to the first molar region (**Figures 5D_1_,D_2_**), was not increased in the *gld* mice (avg*_gld_* = 159, sd*_gld_* = 14) when compared to the wild types (avg_wt_ = 296, sd_wt_ = 47, *p* = 0.0079). The relative decrease of osteoclast number in *gld* mice may result from the reduced bone matrix area in *gld*. In addition, no apparent change in the distribution of apoptotic cells could be detected by the TUNEL assay when comparing mandibular bone of *gld* and wild type mice (**Figures 5G,H**).

**FIGURE 6:**
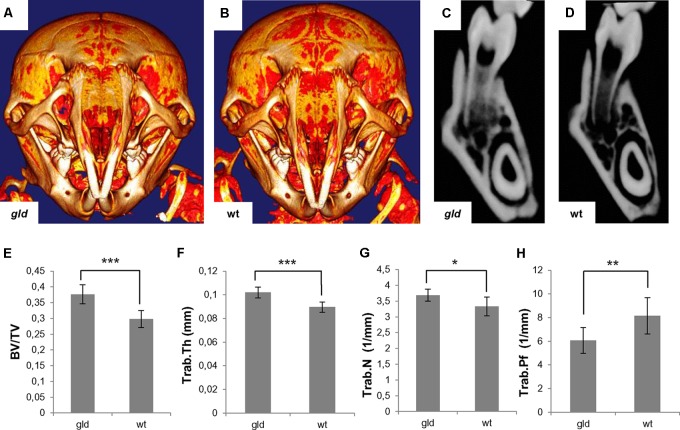
Analysis of postnatal (P24) jaw bone of *gld* mice. Increased mineralization in *gld* **(A)** compared to wild-type mouse **(B)** where bright color shows increased mineralization, frontal section of the mandible of *gld* **(C)** and wild-type mice **(D)**, increased BV/TV ratio in *gld* mice (*n* = 6 animals) compared to wild-type mice (*n* = 6 animals), avg*_gld_* = 0.3763 mm^3^, sd*_gld_* = 0.0301 vs. avg_wt_ = 0.2978 mm^3^, sd_wt_ = 0.0268, *p* = 0.0008, Student’s *t*-test, two-tailed **(E)**, increased Trab.Th in *gld* mice (*n* = 6 animals) compared to wild-type mice (*n* = 6 animals), avg*_gld_* = 0.1020 mm, sd*_gld_* = 0.0046 vs. avg_wt_ = 0.0896 mm, sd_wt_ = 0.0043, *p* = 0.0007, Student’s *t*-test, two-tailed **(F)**, increased Trab.N in *gld* mice (*n* = 6 animals) compared to wild-type mice (*n* = 6 animals), avg*_gld_* = 3.6872 1/mm, sd*_gld_* = 0.1874 vs. avg_wt_ = 3.3277 1/mm, sd_wt_ = 0.2954, *p* = 0.0152, Mann–Whitney test **(G)**, decreased Trab.Pf in *gld* mice (*n* = 6 animals) compared to wild-type mice (*n* = 6 animals), avg*_gld_* = 6.0641 1/mm, sd*_gld_* = 1.0851 vs. avg_wt_ = 8.1548 1/mm, sd_wt_ = 1.5312, *p* = 0.0087, Mann–Whitney test **(H)**. ^∗^*p* ≤ 0.05, ^∗∗^*p* ≤ 0.01, and ^∗∗∗^*p* ≤ 0.001.

MicroCT visualization in postnatal (P24) *gld* mice showed a slightly increased mineralization of the skull bones of *gld* mice compared to wild type animals, including the mandibular region (**Figures 6A,B**). Accordingly, the quantitative analysis showed a statistically significant increase (*p* = 0.0008, avg*_gld_* = 0.3763 mm^3^, sd*_gld_* = 0.0301 vs. avg_wt_ = 0.2978 mm^3^, sd_wt_ = 0.0268) in bone volume/tissue volume (BV/TV) (**Figure 6E**), which was also observed in the frontal sections of the mandibles (**Figures 6C,D**). Further, analysis showed a statistically significant (*p* = 0.0007) increase in trabecular thickness (Trab.Th) in *gld* mice (avg*_gld_* = 0.1020 mm, sd*_gld_* = 0.0046) when compared to wild type (avg_wt_ = 0.0896 mm, sd_wt_ = 0.0043) (**Figure 6F**). Statistically significant (*p* = 0.0152) increase in trabecular number (Trab.N) was observed in *gld* mice (avg*_gld_* = 3.6872 1/mm, sd*_gld_* = 0.1874) in contrast to wild type animals (avg_wt_ = 3.3277 1/mm, sd_wt_ = 0.2954) (**Figure 6G**). The trabecular pattern factor (Trab.Pf) was significantly decreased (*p* = 0.0087) in *gld* mice (avg*_gld_* = 6.0641 1/mm, sd*_gld_* = 1.0851), when compared to wild type mice (avg_wt_ = 8.1548 1/mm, sd_wt_ = 1.5312) (**Figure 6H**). The BMD of *gld* mice showed an increasing trend (avg*_gld_* = 590.779 mg/cm^3^, sd*_gld_* = 85.619) vs. wild type mice (avg_wt_ = 529.221 mg/cm^3^, sd_wt_ = 23.988), but the difference was not statistically significant (*p* = 0.1208, data not shown).

The data indicated specific age-dependent intramembranous mandibular bone phenotype in the *gld* mice.

## Discussion

This report provides data pointing to novel functions of FasL in osteogenesis via modulation of Mmp2 expression in osteoblasts.

When looking at the expression pattern of FasL in the mandibular bone, FasL was already present in the bone forming mesenchymal cells at E13, when the extracellular matrix just became apparent. Expression of FasL was further apparent two days later, when the first complete set of bone cells became established in the mandibular bone (Alfaqeeh et al., 2013). As previously reported (Kawakami et al., 1997; Hatakeyama et al., 2000; Kovacic et al., 2007; Krum et al., 2008), FasL can be found in both, osteoblast and osteoclast cell populations (Park et al., 2005; Kovacic et al., 2007), widely communicating, e.g., via the RANK/RANKL/OPG pathway (Udagawa et al., 1999; Glass et al., 2005).

In order to demonstrate that FasL is not necessarily associated with apoptosis, its tissue distribution was compared to that of TUNEL-positive cells within the forming mandibular bone. The results clearly showed distinct patterns, with FasL being mostly detected in non-apoptotic cells. These *in vivo* results are in agreement with earlier *in vitro* finding, where addition of FasL only weakly increased the proportion of apoptotic cells in both osteoclastogenic and osteoblastogenic cultures (Kovacic et al., 2007).

To further develop the thesis of a non-apoptotic FasL-triggered pathway in osteogenesis, FasL knock-out (*gld*) mice were analyzed. As expected, FasL deficiency did not significantly affect the apoptotic pattern within the early mandibular bone.

To find out if FasL deficiency might impact common osteogenic pathways, the expression screening of major osteogenic markers was performed in the mandibular bone using PCR Arrays. When comparing the data from *gld* vs. wild type mice the most significant decrease was observed for matrix metalloproteinase 2 (*Mmp2*).

Mmp2 is a membrane-associated protein involved in bone remodeling (Kusano et al., 1998), osteoclasts recruitment (Blavier and Delaisse, 1995; Kusano et al., 1998) and angiogenesis (Stetler-Stevenson, 1999). Mmp2 is mostly localized in osteoblasts (Gack et al., 1995). Based on the immunolocalization following the PCR Array analysis, Mmp2, as also FasL, is abundant from the earliest stages of the mandibular formation and is associated with osteoblasts. Interaction of FasL signalization with Mmp2 has been already documented in other system via NFkappaB–TIMP-2 pathway (Wisniewski et al., 2010).

In order to further support the evidence for a FasL effect on Mmp2, an osteoblastic cell line (MC3T3-E1) naturally expressing FasL and Mmp2 (Mizutani et al., 2001; Ozeki et al., 2002) was used to perform a set of functional experiments. Results of the *in vitro* analyses confirmed the engagement of FasL in regulation of *Mmp2* expression as neutralization of FasL by anti-FasL resulted in a significant decrease of *Mmp2* expression and conversely treatment by rmFasL resulted in an increased expression of *Mmp2*. In both cases, the effect of treatment on *Mmp2* expression deepened with time of cultivation. Furthermore, several subsequent rescue experiments validated the mechanism.

The *Mmp2* deficient mice showed a decreased BMD in the endochondral bones of limbs but an increased bone volume in the intramembranous calvaria causing calvarian sclerosis (Inoue et al., 2006). Notably, the phenotype was reported as age-dependent: the bone formation was significantly enhanced in the calvaria bone of the adults but not yet in 7 weeks animals (Inoue et al., 2006).

To search for matching data at the phenotype level in *Mmp2* and *FasL* deficient bones, the investigation was supplemented by histological (prenatal) and microCT (postnatal) analysis of the *gld* mandibular bones, not investigated from earlier research focused on adult endochondral bones (Katavic et al., 2003). Notably, as in *Mmp2* deficient mice, also the *gld* intramembranous bone phenotype displayed age dependent alterations. A mechanism was suggested to explain such phenomenon related to *in vivo* specific osteocyte-osteoblast networks, where the role of sclerostin was also emphasized (Zhao et al., 2000; Inoue et al., 2006). The number of osteocytes increases with age and the sclerotin they secreted inhibits osteoblast proliferation and differentiation (Delgado-Calle et al., 2017). The synergic impact of Sost on the phenotype would be in agreement with our data where FasL deficiency *in vivo* affected not only Mmp2 but also Sost expression.

In summary, these data provide new evidence for non-apoptotic functions of FasL during bone formation. For the first time, FasL expression at early stages of intramembranous ossification was reported and presence of FasL in non-apoptotic cells in the forming mandibular bone was shown. Additionally, it was demonstrated that FasL deficiency impacts the expression of important osteogenic genes including *Mmp2* and jaw bone architecture. And finally, functional experiments proved that FasL regulates *Mmp2* expression in osteoblastic cells. Altogether, since Mmp2 elevated levels were detected in sera of osteoporotic patients (Feng et al., 2016), the present study further strengthens a previously suggested engagement of Fas/FasL system in osteoporosis (Kovacic et al., 2010).

## Author Contributions

ES prepared immunohistochemistry, morphological staining, apoptotic cells and TRAP-positive cells detection, tissue separation, RNA isolation, statistical analysis, and wrote the manuscript. BV performed immunofluorescent detection of markers, PCR Arrays, *in vitro* experiments, qPCR, and manuscript preparation. HL carried out 3D reconstructions and was engaged in manuscript preparation. AP analyzed microCT outcomes, ensured *gld* mice breeding and staging, and manuscript preparation. JS prepared microCT scans and reconstructions. EM was head of the project, responsible for design of study, experimental design, and manuscript preparation.

## Conflict of Interest Statement

The authors declare that the research was conducted in the absence of any commercial or financial relationships that could be construed as a potential conflict of interest.

## Footnotes

1 https://fiji.sc/

## Abbreviations

E: embryonic day
P: postnatal day
